# Multicenter comparison of Etest, Vitek2 and BD Phoenix to broth microdilution for beta-lactam susceptibility testing of *Streptococcus pneumonia*

**DOI:** 10.1007/s10096-024-04847-2

**Published:** 2024-05-27

**Authors:** Steven Martens, Lize Cuypers, Florian Bélik, Pieter-Jan Briers, Pieter-Jan Ceyssens, Olivier Denis, Te-Din Huang, Koen Magerman, Thomas Strypens, Anne-Marie Van den Abeele, Stefanie Desmet, on behalf of the Belgian National Antibiogram Committee

**Affiliations:** 1 National Reference Center for Invasive Pneumococci, University Hospitals Leuven, Herestraat, 49, Leuven, 3000 Belgium; 2 Laboratory of Clinical Microbiology, CHU UCL Namur and Université Catholique de Louvain, Yvoir, Belgium; 3 Laboratory of Clinical Microbiology, Jessa Ziekenhuis, Hasselt, Belgium; 4 Unit of Human Bacterial Diseases, Sciensano, Brussels, Belgium; 5 Laboratory of Clinical Microbiology, AZ Sint-Lucas Ziekenhuis and Universiteit Gent, Gent, Belgium; 6 Laboratory of Clinical Microbiology, Department of Microbiology, Immunology and Transplantation, KU Leuven, Leuven, Belgium

**Keywords:** Beta-lactam, Antimicrobial susceptibility testing, Streptococcus pneumoniae, EUCAST breakpoints

## Abstract

**Purpose:**

To assess performance of Etest®, Vitek®2 and BD Phoenix™ to determine the susceptibility of *Streptococcus pneumoniae* strains to penicillin, ampicillin and cefotaxime.

**Methods:**

Sixty unique *S. pneumoniae* challenge strains were selected to cover a wide range of penicillin, ampicillin and cefotaxime minimal inhibitory concentrations (MICs). Strains were analyzed in four different Belgian laboratories. Etest® benzylpenicillin (BEN), ampicillin/amoxicillin (AMP) and cefotaxime (CTA) (bioMérieux), Vitek®2 AST-ST03 (bioMérieux) and BD Phoenix™ SMIC/ID-11 testing were each performed in two different labs. Results were compared to Sensititre® broth microdilution (BMD) (Thermo Fisher Scientific) results. MIC results were interpreted using EUCAST non-meningitis breakpoints (v 13.0).

**Results:**

Essential agreement (EA) was ≥ 90% for all methods compared to BMD, except for Etest® BEN on Oxoid plate (58.3%), Etest® AMP (both on Oxoid (65.8%) and BD BBL plate (84.2%)). Categorical agreement (CA) for penicillin was only ≥ 90% for Vitek®2, for other methods CA ranged between 74 and 84%. CA for AMP was for all methods < 90% (range 75.8–88.3%) and CA for CTA was between 87 and 90% for all methods except for Etest on Oxoid plate (79.2%).

**Conclusions:**

Our study indicates that Vitek®2 and BD Phoenix™ are reliable for providing accurate pneumococcal susceptibility results for BEN, AMP and CTA. Using Etest BEN or AMP on Oxoid plate carries a risk of underestimating the MIC and should be interpreted with caution, especially when the obtained MIC is 1 or 2 doubling dilutions below the S or R clinical breakpoint.

## Introduction

*S. pneumoniae* is the most common cause of community acquired bacterial pneumonia. *S. pneumoniae* infections can also lead to more severe invasive pneumococcal diseases such as sepsis and meningitis. Treatment of pneumococcal infections is most often based on the use of penicillins or cephalosporins. However, more than 12% of European invasive *S. pneumoniae* are non-wild type for penicillin (BEN) (MIC > 0.06 mg/L). It is therefore important to accurately determine the antimicrobial susceptibility profile of pneumococci to guide the treatment and dosage [1].

The antimicrobial susceptibility testing (AST) of *S. pneumoniae* is complicated by different factors. First, the interpretation of AST results of beta-lactam antibiotics for *S. pneumoniae* depends on the clinical context: meningitis versus non-meningitis. Second, if disk diffusion is used according to the European Committee on Antimicrobial Susceptibility Testing (EUCAST) guidelines, the oxacillin disk screen test can only exclude resistance to all beta-lactams if oxacillin diameter is > = 20 mm. In laboratories primarily using the disk diffusion method for *S. pneumoniae*, MIC testing of BEN is needed for strains with oxacillin diameter < 20 mm. Third, on 21st of November 2019, EUCAST issued a warning against the use of gradient tests for BEN MIC determination in *S. pneumoniae*. A study performed by EUCAST laboratory pointed out that two gradient tests (Etest® from bioMérieux and MRS from Liofilchem) frequently underestimated MIC values by one or more doubling dilutions [2]. This is especially detrimental in the important area close to the R breakpoint, as this may result in very major errors (VME; false susceptibility). For this reason, EUCAST recommended to perform the gold standard broth microdilution method to determine the MIC of BEN. Unfortunately this broth microdilution method has not been introduced (yet) in the majority of the routine European clinical laboratories, mainly due to the fact that no commercial broth microdilution methods as described by EUCAST guidelines are currently available. In addition, in the context of the new in vitro diagnostics regulation (IVD-R), laboratories are not eager to set-up the preparation of Mueller–Hinton broth with 5% lysed horse blood (MH-F) and microtiter plates in the lab.

In most European clinical laboratories, disk diffusion, frequently complemented with gradient diffusion testing and/or commercial automated broth dilution methods are routinely used: e.g. Vitek®2 (bioMérieux) and Phoenix (Becton Dickinson). Regarding the latter commercial automated broth dilution methods, no recent extended evaluation of the performance of MIC testing for *S. pneumoniae* has been described in literature. In this multicenter study, we describe the performance of commercial available methods to determine MICs of penicillin (BEN), amoxicillin/ampicillin (AMP) and cefotaxime/ceftriaxone (CTA). Results of two automated systems, Vitek®2, BD Phoenix™, and one gradient diffusion test, Etest®, on two different, commercially available agars are compared to broth microdilution. Based on these results, we aim to provide guidance to microbiologists, on a suitable alternative method for broth microdilution for the determination of MICs of pneumococci.

All methods were evaluated according to CLSI M52 criteria for categorical agreement (CA), very major error (VME) and major error (ME) rate [3]. ISO document ISO 20776-2:2021 was used for evaluation of essential agreement (EA) and negative and positive bias [4].

## Materials and methods

### Study design

This multicenter laboratory study was set up to compare the MICs obtained by the reference BMD method to those obtained by Vitek®2, BD Phoenix™ and Etest® for BEN, AMP and CTA at four different hospital laboratories in Belgium. Each laboratory tested 60 *S. pneumoniae* strains using one automated system (Vitek®2 or BD Phoenix™) and Etest®. One laboratory additionally performed the BMD method. In Table 1 an overview of the different AST methods performed in the different laboratories is presented.

**Table 1:** AST-methods performed in the different laboratories. The reference method, broth microdilution was performed in laboratory A

Laboratory A	Laboratory B	Laboratory C	Laboratory D
BMD	N/A	N/A	N/A
Vitek®2	BD Phoenix™	BD Phoenix™	Vitek®2
Etest (Oxoid)	Etest (Oxoid)	Etest (BD BBL)	Etest (BD BBL)

### Bacterial isolates

A total of 60 *S. pneumoniae* strains were included in the study: 11 deposited well characterised strains (ATCC 49619 and 10 EUCAST/CCUG strains of *S. pneumoniae)* and 49 clinical strains.

The 49 clinical strains were selected from the repository of the Belgian National Reference Centre (NRC) for *Streptococcus pneumoniae* (invasive) at UZ Leuven, Belgium. Invasive pneumococcal strains isolated between 2020 and 2022 were selected in order to cover a wide range of MICs for BEN, AMP and CTA (respectively from 0.03 to 8 mg/L, from 0.03 to 16 mg/L and from ≤ 0.015 to 8 mg/L). However, most strains were intentionally selected based on MIC values close to the various breakpoints (“challenge strains”).

All the strains were stored in brain heart infusion (BHI) with 10% glycerol at -80 °C and transported in the same tubes on dry ice to the labs. In the labs they were stored at -20/-80 °C until analysis. Strains were subcultured twice on blood agar to check their purity and viability before testing.

Quality control using *S. pneumoniae* strain ATCC 49619 was included in every run when an automated system (Vitek®2 or BD Phoenix™) was used to perform the antibiotic susceptibility tests and as control for Etest® and BMD.

### Antimicrobial susceptibility testing

BMD was performed by making use of a customized antibiotic panel, Sensititre™ BELKUL1 (Thermo Fisher Scientific, Massachusetts, USA). The calling ranges on this plate were ≤ 0.015 to > 16 mg/L for BEN, ≤ 0.015 to > 16 mg/L for AMP and ≤ 0.015 to > 16 mg/L for CTA. CAMHT + LHB broth (Sensititre™ Mueller-Hinton Broth with Lysed Horseblood) was used as described by the manufacturer. Inoculation of the plates was carried out using the Sensititre AIM™ Automated Inoculation Delivery System. Plates were incubated at 35 ± 1 °C in ambient air and bacterial growth was inspected visually after 18 ± 2 h. The reading of the plates was performed using the Sensititre™ Vizion™ Digital MIC Viewing System.

Gradient diffusion tests were performed with Etest® strips (bioMérieux, Marcy l’Etoile, France) on Mueller-Hinton agars with 5% horse blood and 20 mg/L β-NAD (MH-F) from two different suppliers: Oxoid (Thermo Fisher Scientific, product code PB5303A) and BD BBL (BD, product code 257491). Etest® benzylpenicillin, ampicillin and cefotaxime were used on these agars. Bacterial growth was inspected visually after 18 ± 2 h of incubation at 35 ± 1 °C with 5% CO_2_. The MIC was determined to be the value at which the elliptical growth margin intersected the strip. MICs were all measured by one operator.

AST-ST03 cards (BioMérieux, Marcy l’Etoile, France) were used on the Vitek®2 instruments (software version 9.02) and inoculated as recommended by the manufacturer (a McFarland standard of 0.5 to 0.63 in 0.45% sodium chloride using the Vitek DensiChek densitometer). The calling ranges for the Vitek®2 AST-STO3 card were ≤ 0.06 to ≥ 8 µg/mL for BEN, ≤ 0.25 to ≥ 16 µg/mL for AMP and ≤ 0.12 to ≥ 8 µg/mL for CTA.

On the Phoenix platform (Phoenix M50) (software version 7.52B/7.01A) (BD Diagnostic systems, Sparks, MD) the identification (ID) and AST combination panels SMIC/ID-11 were used in this study. The calling ranges for the BD SMIC/ID-11 card were ≤ 0.03 to > 4 µg/mL for BEN, ≤ 0.25 to > 16 µg/mL for AMP and ≤ 0.5 to > 2 µg/mL for CTA.

Each strain was tested by the methods described above from a single fresh overnight culture on a blood agar. The same bacterial suspension adjusted at 0.5 McFarland in a 0.45% saline solution was used for the Vitek®2 and Etest® protocol.

### Statistical analysis

BMD was considered as the reference method for data comparison. The performance of the individual test methods for the different antibiotics was determined based on categorical agreement (CA: percentage of results within the same susceptibility category as the reference method) and essential agreement (EA: percentage of MIC results within a single doubling dilution of MICs as determined by the reference method). Calculation of CA interpretation of all MIC data was done based on EUCAST 2023 breakpoints for indications other than meningitis (e.g. penicillin: S: MIC ≤ 0.06 mg/L; I: MIC between > 0.06 and ≤ 2 mg/L; R: MIC > 2 mg/L) [5].

These EUCAST susceptibility categories (S (susceptible, standard dosing regimen), I (susceptible, increased exposure) and R (resistant)) were also used to calculate the AST error rates. A very major error (VME) was defined as a false susceptible result (S or I reporting instead of R). A major error (ME) was defined as a false resistant result (R reporting instead of S or I). A minor error (mE) was defined as a false result in terms of need for normal or increased exposure in susceptible strains. The VME rates were calculated using the number of resistant strains as denominator and the ME were calculated using the number of susceptible (S + I) strains as denominator [6].

EA and bias were calculated using the ISO 20776-2:2021 document to evaluate the performance of the different methods [4]. Percentages ≥ 90% for CA and EA, VME and ME rate < 3% and a difference for bias ± 30% were considered as acceptable [3, 4].

## Results

### Results of the reference method

The BMD MIC results for BEN, AMP and CTA of the 60 pneumococcal isolates are summarized in Table 2.

**Table 2:** MIC distribution for beta-lactam antibiotics of 60 S. pneumoniae strains based on broth microdilution testing results. The vertical lines indicate the EUCAST clinical breakpoints for non-meningitis. BEN: penicillin; AMP: amoxicillin/ampicillin; CTA: cefotaxime/ceftriaxone

Antimicrobial	MIC (mg/L)										
	≤ 0.015	0.03	0.06	0.125	0.25	0.5	1	2	4	8	16
BEN	0	1	11	4	6	7	7	7	10	7	0
AMP	0	5	8	5	7	6	8	7	3	4	7
CTA	1	4	9	8	6	6	12	10	1	3	0

28% (17/60), 35% (21/60) and 7% (4/60) of strains were resistant to BEN, AMP and CTA, respectively, when tested by BMD and categorized according to the EUCAST 2023 breakpoints for indications other than meningitis.

A total of 120 results per antibiotic and per test method were obtained for Vitek®2, BD Phoenix™, Etest® on Oxoid agar and Etest® on BD BLL agar (Table 3).

**Table 3:** Performance of BD Phoenix^™^, Vitek®2 and Etest® compared to broth microdilution for the determination of susceptibility to penicillin, amoxicillin and cefotaxime of 60 S. pneumoniae strains. Each testing method was performed in 2 different labs

		penicillin						amoxicillin						cefotaxime				
Testing method	EA (%)	CA (%)	VME (*n*)	ME (*n*)	mE (*n*)	bias (%)	EA (%)	CA (%)	VME (*n*)	ME (*n*)	mE (*n*)	bias (%)	EA (%)	CA (%)	VME (*n*)	ME (*n*)	mE (*n*)	bias (%)
BD Phoenix™	**90.8**	82.5	1	15	5	**+ 19.9**	**99.2**	88.3	0	12	2	**+ 7.1**	**100**	87.5	3	0	12	**-24.8**
Vitek®2	**96.6**	**90.0**	6	0	6	**-8.7**	**91.7**	86.7	0	16	0	**+ 18.3**	**99.2**	**90.0**	0	5	7	**+ 7.5**
Etest on Oxoid plate	58.3	74.2	31	0	10	-73.0	65.8	75.8	19	2	8	-72.0	**90.8**	79.2	4	0	21	-35.1
Etest on BD BBL plate	**94.2**	84.2	12	1	6	-**20.4**	84.2	82.5	7	10	4	**-27.7**	**95.0**	87.5	1	2	12	**+ 7.9**

EA, essential agreement; CA, categorical agreement; VME, very major error; ME, major error; mE, minor error

^a^EUCAST 2023 clinical breakpoints for indications other than meningitis were used to interpret the results

^b^CLSI and ISO acceptance rates (EA and CA ≥ 90%, difference bias ± 30%) are in bold

### Quality control

All MICs of the ATCC 49.619 strain were within the QC target ranges for all methods in the different labs. With BMD, Vitek®2 and BD Phoenix™, all measured MICs were within one doubling dilution of the consensus MIC of the CCUG strains. In contrast, with Etest on Oxoid medium, for 6/10 strains (lab B) and 3/10 strains (lab A) the criterium was not met for at least one of the three antibiotics. The MICs found with Etest on BD BBL agar were within the target for all strains in lab C and were out of target for only AMP and BEN each in one of the 10 CCUG strains.

### Penicillin

**Fig. 1:**
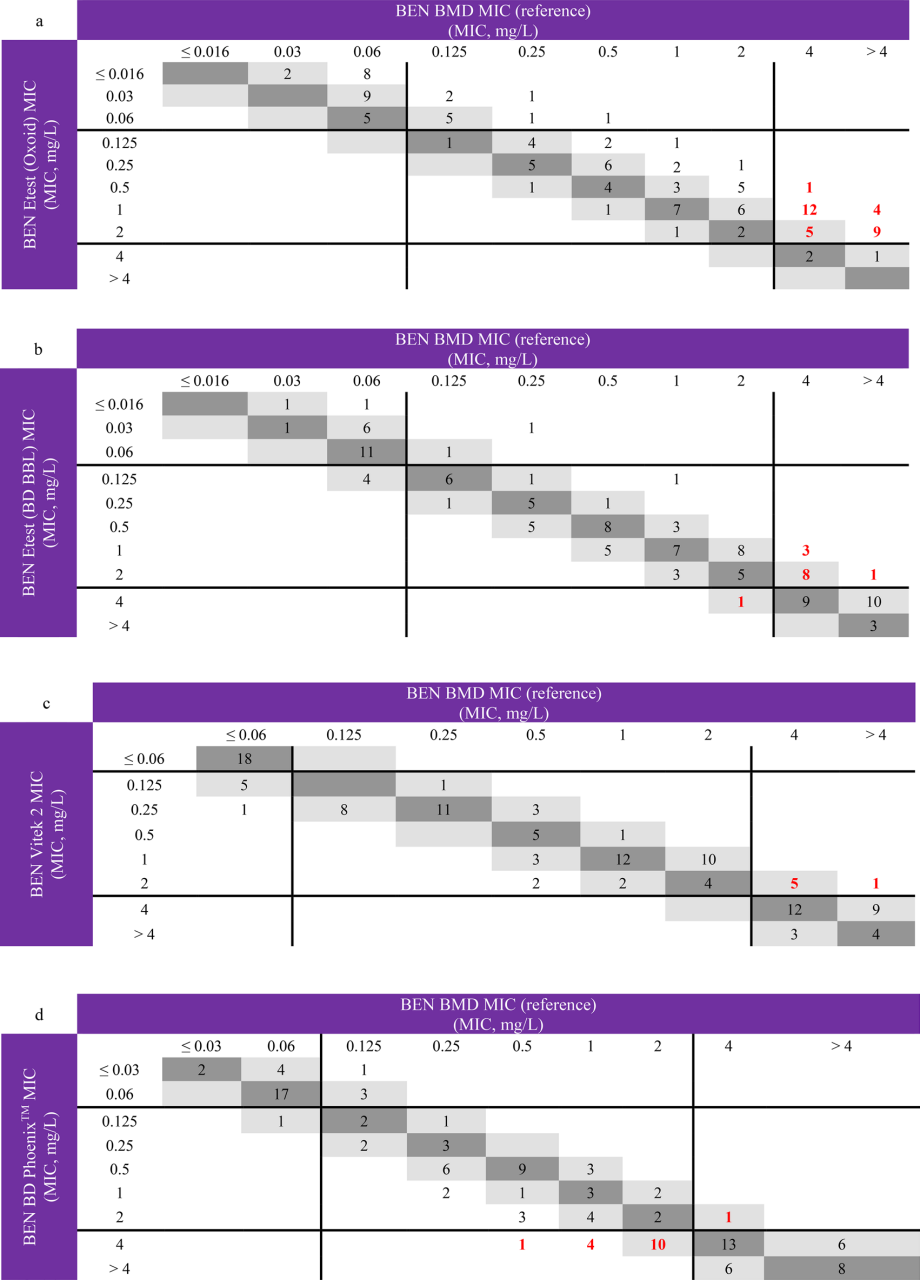
Correlation between BEN MICs for *S. pneumoniae* determined by BMD and by Etest (**a** and **b**), by Vitek®2 (**c**) and by BD Phoenix™ (**d**). The MIC values obtained by BMD are used as reference. The number of strains with MIC corresponding to BMD and 1-log2 dilution are indicated in darker and lighter grey squares, respectively. EUCAST breakpoints for indications other than meningitis are reported as solid black lines. VMEs and MEs are indicated in bold and red

For all methods, except Etest on Oxoid plate, essential agreement for BEN was 90% or more. CA was for all methods lower than the EA, except for the Etest on Oxoid plate, and was highest for Vitek®2 with 90% CA. The categorical errors with Phoenix were mainly due to overestimation of the MIC (ME rate of 17.4%), as for Etest methods there were more underestimations of the MICs resulting in a high VME rate of 91.2% for Etest on Oxoid plate and a VME rate of 35.3% for Etest on BD BBL plate (Fig. 1). This underestimation for the Etest methods is also illustrated by the high percentage of negative bias (-75.8% and -38.3%) on Oxoid and BD BBL plate respectively.

The VMEs for Etest on Oxoid plate were observed in both labs, with 14 VME in lab A and 17 VME in lab B. Also VMEs with Etest on BD BBL plate were observed in both labs: 5 VME in lab C and 7 VME in lab D.

### Amoxicillin/Ampicillin

Essential agreement was only below 90% when using a gradient diffusion test. The automated systems had an EA agreement of 91.7% (Vitek®) and 99.2% (BD Phoenix™). Similarly to Etest PEN, Etest AMP tended to provide low MIC values in comparison to BMD, as illustrated by the high percentage of negative bias (-76.7% and -47.5%) on Oxoid and BD BBL plate respectively. Categorical agreement was below 90% for all methods, with the lowest CA for Etest on Oxoid plate (75.8%). The low CA for Etest on Oxoid plate was due to 19 VME, 2 ME and 8 mE. The number of VME errors was higher in lab B (*n* = 13) than lab A (*n* = 6). For the two automated methods (Phoenix (CA 88.3%) and Vitek®2 (CA 86.7%)) and Etest on BD BBL plate (CA 82.5%), the low CA was mainly due to major errors.

### Cefotaxime

EA was high (> 90%) for all methods. CA was between 87 and 90% for all methods, except for Etest on Oxoid plate with a CA of 79.2%, mainly caused by a high number of minor errors (*n* = 21). VME errors were observed for every method, except for Vitek®2. In contrast Vitek®2 had the highest number of ME (*n* = 5).

## Discussion

In this study we confirmed the underestimation of BEN MIC with Etest as described in a warning and previous study performed in the EUCAST laboratory [2]. In addition, we observed also for AMP a low EA and CA, primarily when the Etest method was performed. The low EA and CA were more pronounced for Etest on Oxoid plate than on BD BBL plate. CTA Etest results were more in accordance with the BMD results and results of the automated methods, with acceptable EA for all methods, including Etest.

Our findings regarding performance of Etest and Vitek®2 are in line with the results of a study from Charles et al. They tested 91 *S. pneumoniae* strains with Etest, M.I.C.E., Vitek®2 and Sensititre and results were compared to BMD, the golden standard [7]. In contrast to our study, CLSI M100-S25 non-meningeal breakpoints were used. EA for Etest BEN, AMP and CTA was 95%, 74% and 98% with a corresponding CA of 90%, 84% and 88% respectively. They also observed Etest BEN and AMP MICs of 1 or more doubling dilutions below the MIC obtained by BMD. EA for Vitek®2 BEN and CTA were 89% and 93% with a corresponding CA of 90% and 85% respectively when applying non-meningeal breakpoints.

The performance of the Etest differed on the two media tested, with a lower performance on the Oxoid agar compared to the BD BBL agar. These observations are in line with previous studies of the EUCAST laboratory, underscoring the impact of the brand of the agar plates on the performance of disk diffusion testing [8]. Moreover interlaboratory differences were observed between labs using the same plates. Labs used reagents and plates from the same batch, and these were transported to the labs at the same moment in the same conditions. For this reason, we assume that the interlaboratory variation is not a problem of different technical performance, but probably rather a problem due to the subjective reading of the Etest, which is complicated by a zone of alpha-hemolysis.

In the same study we evaluated the performance of two commercialy available automated AST methods that are widely used in Belgian and European clinical laboratories. BD Phoenix™ and Vitek®2 had high EA for BEN and CTA, while only for AMP EA was < 90%. Depending on the antibiotics tested we observed that with BD Phoenix™ MICs of BEN and AMP were in some strains overestimated, leading to MEs. With Vitek®2 we observed underestimations of BEN MIC in some strains, resulting in VMEs and overestimations of AMP and CTA MICs leading to MEs. Probably the strain selection played a role in the low CA (≤ 90%) for all methods, as we selected pneumococcal strains with MICs close to the clinical breakpoints. In Europe, about 84% of the pneumococcal strains are wild type for betalactam antibiotics [1] in contrast to only 20% of the strains in this study. As a result, the obtained data (percentages of agreement) are better viewed in a comparative manner rather then seen as a fixed and absolute interpretation. Taking into account the high EA compared to BMD, these automated AST methods seem to be an alternative method for MIC determination in routine clinical laboratories that have no BMD in the laboratory available.

We observed that ATCC 49619 is not able to detect potential quality problems for beta-lactam antibiotics, and it is therefore highly recommended that laboratories use other well validated strains during implementation validation of AST methods for *S. pneumoniae* testing, such as the CCUG-EUCAST strains.

A major strength of our study is that it was performed in different laboratories and with the same batch of bacterial strains and reagents, i.e. the same lot of Etest strips and AST panels. However there are some limitations. First, we used Sensititre BMD as the gold standard method. This resulted in the use of Mueller–Hinton broth with 5% lysed horse blood as recommended by ISO and CLSI instead of Mueller-Hinton broth with 5% lysed horse blood and 20 mg/L beta-NAD (MH-F broth) recommended by EUCAST. However, for all CCUG strains the BMD MICs were within the targets described by EUCAST. Second, we included 60 strains in the panel with a high proportion of non-wild type strains, probably resulting in lower performance for all tested methods. Third, we tested 2 different pre-poured MH-F media, but the conclusions regarding Etest may be not applicable for Etest on other MH-F agar. Fourth, the interpretation of CA is based on the susceptibility testing categories S, I and R according to EUCAST breakpoints. The definition and meaning of the I category however changed on the first of January 2019 (EUCAST breakpoint table v.9.0) from “Intermediate” to “Susceptible, increased exposure”. As a result, previous CA interpretations (prior to 2019) were based on the historical definition of I. To the best of our knowledge EUCAST has not published any guidance document yet on how to apply CA interpretation under the new I definition.

## Conclusion

Our findings indicate that Vitek®2 and BD Phoenix™ provide accurate susceptibility results for BEN, AMP and CTA based on high EA compared to BMD. In contrast, with Etest on Oxoid agar and to a lesser extent Etest on BD BBL agar, there is a risk of underestimation of the MICs of BEN. Additionally, interlaboratory variation for Etest methods and low EA and CA for AMP Etest on Oxoid plate was observed.
